# A Diet Rich in Fish Oil and Leucine Ameliorates Hypercalcemia in Tumour-Induced Cachectic Mice

**DOI:** 10.3390/ijms20204978

**Published:** 2019-10-09

**Authors:** Rogier L.C. Plas, Mieke Poland, Joyce Faber, Josep Argilès, Miriam van Dijk, Alessandro Laviano, Jocelijn Meijerink, Renger F. Witkamp, Ardy van Helvoort, Klaske van Norren

**Affiliations:** 1 Division of Human Nutrition and Health, Wageningen University, 6700 EV Wageningen, The Netherlandsmieke.poland@wur.nl (M.P.); jocelijn.meijerink@wur.nl (J.M.); renger.witkamp@wur.nl (R.F.W.); 2 Danone Nutricia Research, Nutricia Advanced Medical Nutrition, 3584 CT Utrecht, The Netherlands; j.faber@dz.nl (J.F.); miriam.vandijk@nutricia.com (M.v.D.); ardy.vanhelvoort@danone.com (A.v.H.); 3 Cancer Research Group, Departament de Bioquímica i Biologia Molecular, Facultat de Biologia, Universitat de Barcelona, 08193 Barcelona, Spain; jargiles@ub.edu; 4 Department of Clinical Medicine, University La Sapienza, 185 Rome, Italy; alessandro.laviano@uniroma1.it; 5 NUTRIM School of Nutrition and Translational Research in Metabolism, Faculty of Health, Medicine, and Life Sciences, Maastricht University, 6229 ER Maastricht, The Netherlands

**Keywords:** hypercalcemia, PTHrP, cachexia, fish oil, leucine

## Abstract

Background: Dietary supplementation with leucine and fish oil rich in omega-3 fatty acids docosahexaenoic acid (DHA) and eicosapentaenoic acid (EPA) has previously been shown to reduce cachexia-related outcomes in C26 tumour-bearing mice. To further explore associated processes and mechanisms we investigated changes in plasma Ca^2+^ levels, the involvement of parathyroid hormone related protein (PTHrP), and its possible interactions with cyclooxygenase 2 (COX-2). Methods: CD2F1 mice were subcutaneously inoculated with C26 adenocarcinoma cells or sham treated and divided in: (1) controls, (2) tumour-bearing controls, and (3) tumour-bearing receiving experimental diets. After 20 days, body and organ masses and total plasma Ca^2+^ levels were determined. Furthermore, effects of DHA, EPA and leucine on production of PTHrP were studied in cultured C26 cells. Results: The combination of leucine and fish oil reduced tumour-associated hypercalcemia. Plasma Ca^2+^ levels negatively correlated with carcass mass and multiple organ masses. DHA was able to reduce PTHrP production by C26 cells in vitro. Results indicate that this effect occurred independently of COX-2 inhibition. Conclusion: Our results suggest that cancer-related hypercalcemia may be ameliorated by a nutritional intervention rich in leucine and fish oil. The effect of fish oil possibly relates to a DHA-induced reduction of PTHrP excretion by the tumour.

## 1. Introduction

Cancer-related hypercalcemia is seen in up to 30% of patients with malignancies [1]. Very often, this is accompanied by increased bone resorption [2]. Calcium plays a vital role in many different physiological functions, for example in the contraction of all muscle cell types and neuronal signalling. In healthy individuals, the plasma concentration of calcium is tightly regulated by the interplay of parathyroid hormone (PTH), vitamin D and calcitonin [2]. In patients with malignant disorders, calcium balance is often disrupted, reflected by elevated calcium plasma levels [1]. Calcium is partly bound to albumin and its plasma levels are either expressed as albumin corrected levels (common in clinical practice) or as total Ca^2+^ levels (free + bound). Hypercalcemia is defined in patients as mild for levels between 10.5 and 11.9 mg/dL (2.6–2.9 mmol/L), as moderate between 12 and 13.9 mg/dL (3.0–3.4 mmol/L), and as severe above 14 mg/dL (3.5 mmol/L) serum total Ca^2+^ [1,3]. The main cause of malignancy-related hypercalcemia is an imbalance in bone formation and resorption [3].

The most prevalent clinical symptoms with hypercalcemia relate to neurologic, psychiatric, gastrointestinal, cardiovascular and renal abnormalities [2,3,4]. Neurologic and psychiatric symptoms include fatigue, lethargy, musculoskeletal pain, depression, and even coma. Reduced motility of the gastrointestinal tract can cause constipation and reduced appetite. Cardiovascular symptoms include cardiac arrhythmias and hypertension. Moreover, renal failure is frequently present in hypercalcemia. Symptoms of hypercalcemia are frequently seen in cancer patients. For example, in multiple myeloma patients, serum calcium levels were an independent predictor of quality of life, fatigue and physical functioning [5].

There are three proposed mechanisms by which malignancies can affect the balance between Ca incorporation in bone and its resorption [2]. The first is associated with increased degradation of bone by osteoclasts, which become activated by factors secreted by metastases or primary tumours in or close to the bone. A second mechanism involves increased levels of inflammatory mediators like interleukin 6 (IL-6), prostaglandin E2 (PGE-2), and of PTH-related protein (PTHrP), which directly cause increased breakdown of bone. A third possibility is based on the connection to coexisting primary hyperparathyroidism. Of these three proposed mechanisms, the one involving PTHrP secreted by the tumour is considered the most prominent and responsible for 80% of all malignancy-related hypercalcemia patients [4].

PTHrP is a protein between 139 and 173 amino acids in size. Its N-terminal shows homology with PTH. PTHrP is produced in low concentrations by practically all tissues. PTHrP produced by the tumour can bind and activate the PTH receptor leading to increased bone demineralization and increased renal reabsorption of calcium [6]. Both these processes contribute to the increase in plasma calcium levels. Bone resorption can lead to TGFβ release, which in turn stimulates PTHrP secretion by tumour cells, thus initiating a vicious cycle [6,7]. The elevation of PTHrP in C26 adenocarcinoma cells has been reported to depend on an increase in cyclooxygenase 2 (COX-2) activity, which in parallel also results in an elevated PGE-2 production [8]. Apart from its effects on bone calcium turnover, PTHrP also directly stimulates muscle wasting and adipose tissue browning [9]. Possibly related to this, serum PTHrP levels were found to be predictive of weight loss in cancer patients independently of hypercalcemia, inflammation and tumour burden [10]. Together, these findings suggest an overlap between features of cancer cachexia and malignancy-related hypercalcemia, two clinically relevant paraneoplastic syndromes. In order to investigate whether this overlap would also apply to possible intervention strategies for cachexia, the present study was initiated. This study investigated the effects of a diet enriched in leucine, protein and fish oil on PTHrP related changes of plasma Ca levels in a tumour-induced cachexia model. In the same animals, we previously showed significant improvement of cachexia-related outcomes in those mice that had received such specific nutritional combination (SNC) [11]. Starting from the hypothesis that supplementation of fish oil and leucine might also reduce plasma Ca^2+^ levels in tumour-bearing animals, we further investigated the involvement of PTHrP and COX-2 in the regulation of Ca^2+^ levels by docosahexaenoic acid (DHA) and eicosapentaenoic acid (EPA), respectively. To this end, we performed a series of in vitro experiments with C26 cells.

## 2. Results

### 2.1. Effect of Leucine and Fish Oil in Vivo in Experiment A,B

#### 2.1.1. Calcium Levels and Their Correlation with Carcass Mass, Organ Masses and EDL Muscle Function.

**Figure 1:**
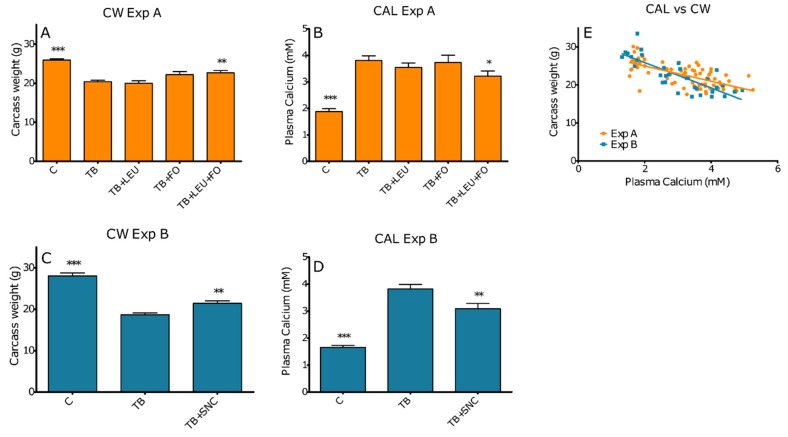
Effect of leucine (LEU), fish oil (FO) and a combination of leucine and fish oil on carcass weight and plasma Ca^2+^ levels (**A**,**B**); and effect of a specific nutritional combination (SNC) containing added fish oil and leucine on carcass weight and plasma Ca^2+^ levels (**C**,**D**). Data represent mean ± SEM. Correlation between carcass weight and plasma Ca^2+^ levels (Pearson r = −0.6684 [Experiment A] and −0.8097 [Experiment B], both with *p* < 0.0001) (**E**). *, ** and *** represent significant differences with tumour-bearing (TB) group (respectively, *p* < 0.05, *p* < 0.01 and *p* < 0.001).

We determined whether cachexia-associated outcomes might be related to plasma Ca^2+^ levels. As previously published, carcass mass was decreased, as seen in Figure 1A,C, in tumour-bearing (TB) mice, compared to the carcass mass of control mice which was improved to a certain extent by supplementation of fish oil and leucine or the SNC [11]. Our additional analysis showed a two-fold increase in plasma Ca^2+^ levels in the tumour-bearing animals as seen in Figure 1. The combination of leucine and fish oil was able to significantly reduce the tumour-induced hypercalcemia, whereas the individual components could not reduce hypercalcemia as seen in Experiment A, Figure 1B. Similar reductions in plasma Ca^2+^ levels were found in animals supplemented with the complete SNC containing high protein, leucine, fish oil and oligosaccharides as seen in Experiment B, Figure 1D. Moreover, plasma Ca^2+^ levels were negatively correlated with carcass weight and with multiple organ masses as seen in Figure 1E and Table 1. Muscle function of mEDL was determined ex vivo in an organ bath. For all frequencies at which tetanus could be obtained (>83 Hz), muscle function parameters (maximal force, maximal contraction velocity and maximal relaxation velocity), correlated negatively with calcium levels (R below −0.7 and *p* < 0.05) as seen in Table 2. When the muscle function parameters, maximal force, maximal contraction velocity and maximal relaxation velocity were corrected for muscle mass, negative correlations remained, though R values were less (between −0.35 and −0.7), and significant levels were obtained for all parameters for frequencies ≥100 Hz.

#### 2.1.2. Plasma PGE-2 and Tumour PTHrP

**Figure 2:**
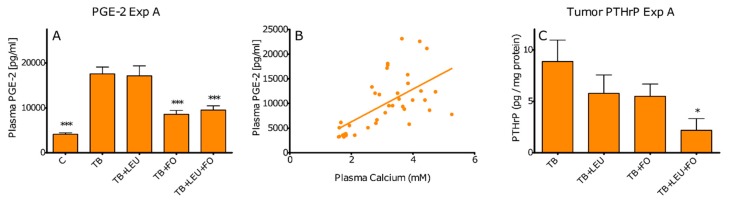
Effect of leucine (LEU), fish oil (FO) and a combination of leucine and fish oil on plasma PGE-2 (**A**); correlation between plasma PGE-2 and plasma Ca2+ levels (Pearson r = 0.6062 with *p* < 0.0001) (**B**); and tumour PTHrP (**C**). Data represent mean ± SEM. *, ** and *** represent significant differences with TB group (respectively *p* < 0.05, *p* < 0.01 and *p* < 0.001).

Inflammatory mediators like PGE-2 and PTHrP have been reported to play a potential role in the onset of hypercalcemia. Plasma PGE-2 levels were significantly increased in TB compared to control mice as seen in Figure 2B. This increase was reduced upon supplementation of fish oil either with or without added leucine. Plasma Ca^2+^ and PGE-2 levels correlated significantly (Pearson r = 0.6062 with *p* < 0.0001) as seen in Figure 2F. Tumour PTHrP levels were significantly lower in TB animals that had received diets enriched with fish oil and leucine compared to TB animals without supplementation as seen in Figure 2C. Tumour PTHrP levels did not correlate with plasma Ca^2+^ levels. However, it should be noted that there were no PTHrP levels determined in control animals since they have no tumour.

### 2.2. Effect of Leucine and Fish Oil in Vitro in Experiment C–E

#### 2.2.1. Supplementation of C26 Cells with Nutritional Components used in Vivo in Experiment C,D

**Figure 3:**
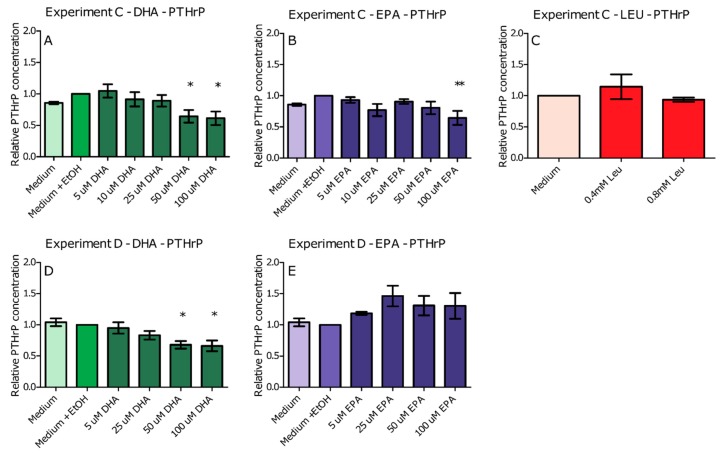
Effect of supplementation of C26 cells with docosahexaenoic acid (DHA) (**A**,**D**); eicosapentaenoic acid (EPA) (**B**,**E**); and leucine (LEU) (**C**) on PTHrP levels on low (**A**–**C**); and high (**D**,**E**) number of cells. Data represent mean ± SEM. *, ** and *** represent significant differences with vehicle control (respectively, *p* < 0.05, *p* < 0.01 and *p* < 0.001).

To determine possible mechanisms behind the effects of the nutritional supplementation with leucine and fish oil in C26 mice, a sequence of in vitro experiments was performed. In Experiment C, small numbers of C26 cells were incubated with omega-3 fatty acids EPA or DHA, or leucine added to the medium and PTHrP production was measured. Experiments showed that DHA and EPA at a concentration of 50 μM (DHA), 100 μM (DHA) and 100 μM (EPA) significantly reduced C26 PTHrP production by 36%, 39% and 35%, respectively, as seen in Figure 3A,B. Leucine had no effect on PTHrP production in vitro as seen in Figure 3C. None of the components had any effect on viability or toxicity in the concentrations tested as seen in Figure S1. Given that DHA and EPA were found to be the most potent in reducing PTHrP, these were incorporated into the next experiments. To test the consistency of the findings and to mimic the effects of the potent components DHA and EPA on the tumour, we tested the effects on cells with a higher confluence in Experiment D. The effect of EPA was no longer present. The effect of DHA, however, was reproducible in these confluent cells with reductions of 32% and 34% at 50 μM DHA and 100 μM DHA, respectively, as seen in Figure 3D,E.

#### 2.2.2. Possible Involvement of COX-2 in the Effect of Fish Oil in Experiment E

**Figure 4:**
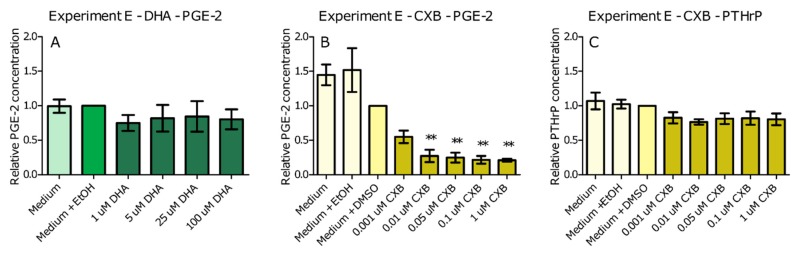
Effect of supplementation of C26 cells with DHA (**A**); and CXB (**B**) on PGE-2 levels; and effect of supplementation of CXB (**C**) on PTHrP levels. Data represent mean ± SEM. *, ** and *** represent significant differences with vehicle control (respectively, *p* < 0.05, *p* < 0.01 and *p* < 0.001).

DHA is known to reduce COX-2 activity [12], and PTHrP production is known to be stimulated upon stimulation of COX-2 [8]. Therefore, to further elucidate the possible mode of action of DHA, we determined whether COX-2 was involved in the PTHrP reducing effects of DHA in Experiment E. This was done by measuring PGE-2 production upon incubation of C26 cells with DHA. As a positive control, the specific COX-2 inhibitor celecoxib (CXB) was used. Results showed that DHA was not able to reduce PGE-2 production in C26 cells where CXB reduced PGE-2 levels with 73% at a dose of 0.01 μM, as seen in Figure 4A,B. Moreover, PTHrP production of C26 cells was not reduced when COX-2 activity was inhibited by incubation with CXB as seen in Figure 4C. This indicates that the effect of DHA was possibly not mediated via the enzyme COX-2. In Experiment D,E, none of the conditions resulted in an increased toxicity, as measured by LDH as seen in Figure S2.

## 3. Materials and Methods

### 3.1. In Vivo Experiments

#### 3.1.1. Animals and Experimental Diets

For the present study, we started our research by analysing archived materials from a previous in vivo study, which made it possible to limit new animal research. That study investigated the effects of a specific nutritional combination of oligosaccharides, high protein, leucine, and fish oil on muscle and daily activity [11]. Experimental procedures have been described in detail in the previous paper. Briefly, male CD2F1 mice aged 6–7 weeks were divided into weight-matched groups: (1) control mice receiving control diet high in protein (C), (2) tumour-bearing mice receiving the same control diet (TB), and (3) tumour-bearing mice receiving experimental diets. In Experiment A, the effect of additional leucine (LEU) and fish oil (FO) on top of the high protein diet was tested. In Experiment B, the specific nutritional combination (SNC) of oligosaccharides, high protein, leucine and fish oil was used. All diets used in the different experiments were iso-caloric, by exchanging additional protein or fat with carbohydrates. In Experiment A, AIN93-M, supplied as pellets, was used as control diet, and for the experimental groups the specific nutritional components were added. In Experiment B, a more human-like control diet was used which was iso-caloric and isonitrogenous to the diet used in Experiment A. Both the control diet as the iso-caloric experimental SNC in Experiment B were supplied as dough. Experimental procedures were approved by the Animal Ethics Committee (DEC consult, Bilthoven, The Netherlands) and complied with the principles of good laboratory animal care (DEC/DAN100).

#### 3.1.2. Experimental Protocol

Murine C26 adenocarcinoma cells were cultured and inoculated as reported previously [11,13]. Briefly, under general anaesthesia (isoflurane/N_2_O/O_2_), tumour cells (5 × 10^5^ cells in 0.2 mL) were inoculated subcutaneously into the right inguinal flank of the mice. Control (C) animals received a sham injection with 0.2 mL HBSS. Animals were weighed and anaesthetized (isoflurane/N_2_O/O_2_) at day 20 after tumour inoculation. Skeletal muscle and internal organs were dissected and weighed. Carcass mass was calculated by subtracting tumour mass from body mass. Ex vivo EDL muscle function was measured at different frequencies ranging from 20 to 167 Hz. Tetanus was reached at frequencies ≥83 Hz.

#### 3.1.3. Plasma PGE-2 and Tumour PTHrP in Experiment A

In animals from Experiment A, plasma PGE-2 was measured using a commercial anti-PGE-2 rabbit polyclonal antibody-based direct enzyme immunoassay (Oxford Biomedical Research, Oxford, MI, USA) according to the manufacturer’s protocol. PTHrP levels in the tumour of animals in Experiment A were measured using a quantitative PTHrP enzyme-linked immunosorbent (ELISA) assay kit (USCN Life Science Inc., Wuhan, Hubei, China) according to the manufacturer’s protocol. PTHrP levels were expressed as amount per milligram of protein as determined using a Pierce^TM^ BCA protein kit (Thermo Fisher Scientific, Rockford, Illinois, USA). Unfortunately, the amount of stored plasma material was insufficient to determine plasma PTHrP levels.

#### 3.1.4. Plasma Calcium in Experiment A,B

To investigate if the elevation of calcium levels was linked to body and organ masses and PGE-2 levels, total plasma Ca^2+^ levels (free calcium + calcium bound to albumin) were determined calorimetrically at the Clinical Chemistry Laboratory Hospital Reinier de Graaf (Delft, the Netherlands).

### 3.2. In Vitro Experiments

To determine possible underlying mechanisms, we set up a series of in vitro experiments. First, we tested the main components present in the diets used in the mouse studies on a low number of tumour cells, as seen in Experiment C. As a control, viability and toxicity assays were performed to rule out potentially toxic effects of components in the concentrations tested. Subsequently, we continued with the most potent compounds on a larger number of cells, as seen in Experiment D, to test their potency in situations more resembling the in vivo situation (i.e., the tumour consists of a very large number of cells). Lastly, we examined a possible mechanism of action of the most potent compound, as seen in Experiment E, by assessing the possible involvement of COX-2 by using a specific COX-2 inhibitor. In Experiment D,E, only toxicity assays were performed.

#### 3.2.1. Culture of Murine C26 Cells

All in vitro experiments were performed using murine C26 tumour cells (American Type Culture Collection; ATCC, Teddington, UK). Cells were cultured in DMEM with 10% heat-inactivated foetal bovine serum at 37 °C in a 5% CO_2_ humidified air atmosphere. For supplementation in Experiment C,D,E, cells were seeded in a 24-wells plate. For Experiment C, 25,000 cells were seeded leading to a confluence of 10–20% after 24h with ample cell–cell contact. For Experiment D,E, 250,000 cells were seeded leading to a confluence of 80–90% after 24h with a high degree of cell–cell contact.

#### 3.2.2. PTHrP and PGE-2 Production upon Supplementation of C26 cells with DHA, EPA, Leucine and CXB

After 24 h of incubation, culture medium from each well was removed and C26 cells were supplemented with test compound or vehicle control. Supplementation for 24h was performed with different concentrations of either docosahexaenoic acid (DHA, 22:6*n-3*; Sigma-Aldrich, Sigma-Aldrich Chemie GmbH, Schnelldorf, Germany), eicosapentaenoic acid (EPA, 20:5*n-3*; Sigma-Aldrich Chemie GmbH), leucine (Sigma-Aldrich Chemie GmbH), or celecoxib (CXB, Sigma-Aldrich Chemie GmbH). Ethanol was used as solvent for ω-3 PUFAs (DHA and EPA), DMSO was used as solvent for CXB and leucine was dissolved in PBS. In all cases, final ethanol/DMSO concentration never exceeded 0.1% v/v. All experiments were performed at least three times, and each condition was done in duplicate or triplicate. After 24h of supplementation, supernatant was removed for analysis of PTHrP and PGE-2. PTHrP production in C26 cell supernatant was measured using a commercially available ELISA (USCN Life Science Inc., Wuhan, Hubei, China) according to the manufacturer’s protocol. PGE-2 production was measured using a commercially available monoclonal ELISA kit (Cayman chemical, Ann Arbor, Michigan, USA).

#### 3.2.3. Viability and Cytotoxicity

For Experiment C, cell viability was measured with the Cell Proliferation (XTT) Kit II (Roche, Basel, Switzerland), according to the manufacturer’s protocol. Cells seeded in a 24-wells plate were incubated for 30 to 90 min with the XTT reagent mix. After incubation, 100 uL supernatant was transferred to a 96-wells plate and absorbance was measured at 450 nm using an ELISA plate reader. As a negative control, cells were treated with Triton X100 which resulted in total cell lysis.

Cytotoxicity was measured for each experiment with the Cytotoxicity Detection (LDH) Kit (Roche, Basel, Switzerland), according to the manufacturer’s protocol. This kit measures the relative LDH content present in the supernatant, which reflects cytotoxicity. Supernatant of the cells seeded in a 24-wells plate was transferred to a 96-wells plate and used to measure LDH content. Absorbance was measured at 492 nm using an ELISA plate reader. As a positive control, cells were incubated with Triton X100 which results in total cell lysis. Viability and cytotoxicity results are reported in Figures S1 and S2.

### 3.3. Statistics

All data are expressed as mean ± SEM. Statistical analyses were performed using GraphPad Prism 5 (GraphPad Software Inc., La Jolla, California, USA). In Experiment A, different batches of animals were used. Therefore, for all parameters, it was defined that combining the data was allowed, meaning no interaction between groups and experiments was present. For all the in vivo experiments, comparisons were made using an analysis of variance (ANOVA) with a Dunnett’s multiple comparison test with the tumour-bearing group without nutritional supplementation as reference. Differences were considered significant at a *p*-value < 0.05. Correlations were calculated and Pearson correlation coefficients with a *p*-value < 0.05 were considered significant. For the in vitro experiments, comparisons were made using an ANOVA with a Dunnett’s multiple comparison test with the vehicle control as reference. Differences were considered significant at a *p*-value < 0.05.

## 4. Discussion

In line with our hypothesis and the available literature [14,15], tumour-bearing cachectic mice showed hypercalcemia. Our data also demonstrate that plasma Ca^2+^ levels correlated negatively with carcass mass and several organ masses. In addition, Ca^2+^ levels positively correlated with plasma PGE-2 levels. Moreover, the nutritional combination reduced elevated plasma PGE-2 levels and PTHrP levels in tumour tissue.

Only a few studies on the relation between fish oil, or its main components EPA and DHA, and tumour-associated hypercalcemia have been reported in the literature. In a 1984 study, mice bearing the prostaglandin producing HSDM1 fibrosarcoma, received menhaden fish oil high in EPA (EPA:DHA ratio of 2.4:1). This intervention caused a reduction of plasma Ca^2+^ levels in tumour-bearing mice [16]. Since hypercalcemia is related to bone mineral density and a reduction of bone mineral density is a well-known feature of the C26 model, we also investigated the literature on reported associations between fish oil treatment and bone mineral density. However, a systematic review on ω3 fatty acids and osteoporosis concluded that clear conclusions are difficult to make, due to the small number of studies and modest sample sizes [17]. In breast cancer survivors, high doses of EPA and DHA were reported to reduce bone resorption [18]. Moreover, fish oil was found to prevent breast cancer cell metastasis to bone and osteolytic lesions in a human mouse xenograft model [19].

In our study, decreased Ca^2+^ levels were only observed when leucine and fish oil were combined. Interestingly, this suggests that leucine might have an additional favourable effect, but only in the presence of fish oil. Leucine has been reported to influence insulin sensitivity of tissues and to increase insulin release [20]. In addition, insulin seems to have an anti-phosphaturic effect [21]. It was reported that in vivo administration of PTH induced a decline in tubular reabsorption of phosphate, which was reversed by superimposition of an euglycemic hyperinsulinemia within the physiologic range [21]. The effect of leucine on hypercalcemia might, therefore, be explained by increased insulin release, leading to increased renal absorption of phosphate.

In our study, dietary fish oil alone, or combined with leucine, reduced plasma PGE-2 levels. Omega-3 PUFAs have been evaluated in various clinical studies for their immunomodulatory capacity [22]. Already after a week of intervention with a medical food high in protein, leucine, fish oil, and specific oligosaccharides, plasma PGE-2 levels decreased in cancer patients receiving radiotherapy [23]. The SNC tested in our study has also been shown to exert immunomodulatory effects by reducing IL-6, TNF-α, IL-4, and PGE-2 plasma levels in the C26 model [24]. Another study reported a decrease in PGE-2 levels in a small intestinal tumour of Apc^Min/+^ mice upon feeding with a high fat diet rich in fish oil [25]. Eicosapentaenoic acid has been reported to decrease the pro-inflammatory steady state by reducing the levels of TNF-α, IL-1β, IL-6, and IL-8 in serum or plasma [26]. Moreover, EPA induces a shift in the pattern of prostaglandins produced by COX-2, from PGE-2 production with arachidonic acid (AA) as substrate, to relatively more PGE-3 produced with EPA as substrate [27,28]. These results are all in line with our findings. However, there is also some conflicting evidence showing that both DHA and EPA can stimulate PGE-2 production in both ScGT1 neuronal cells, raw 264.7 murine macrophages and human primary monocyte-derived macrophages [29,30].

The negative correlations between carcass weight and calcium levels, and those between muscle weight and calcium indicate that the cachexia and hypercalcemia might be linked in our model. It is, however, difficult to indicate to what extent they are related. With regards to muscle function, it is, however, possible to correct for muscle mass. We revealed a high inverse correlation between muscle function and hypercalcemia for maximal force, contraction velocity and relaxation velocity. This correlation remained after correction for muscle mass, though with lower R values, indicating that there seems to be a mass-independent compound in the relationship between calcium levels and loss of muscle function as part of the cachexia syndrome.

Previous studies report that hypercalcemia in the C26 model is mediated by IL-6 and PTHrP [31]. We realize the limitations of this study as we were unable to measure either PTHrP or IL-6 in plasma. From literature, we know that the same SNC is able to reduce IL-6 plasma levels in the C26 model [24]. Moreover, our results showed that the nutritional combination of fish oil and leucine reduced PTHrP levels in the tumour. Elevation of PTHrP in malignancy is thought to be mediated by an increase in COX-2 activity [8]. Therefore, the strong positive correlations of Ca^2+^ levels with PGE-2, a major COX-2-mediated inflammatory mediator, might suggest that PTHrP is also involved in the induction of hypercalcemia. To test this hypothesis, we performed a sequence of in vitro experiments.

Our results showed that among the nutrients tested in vivo, EPA and DHA seemed most potent when a low number of cells was used, as seen in Experiment C. Interestingly, leucine did not have any effect in vitro, whereas in vivo a combination of fish oil and leucine was needed to reduce Ca^2+^ and tumour PTHrP. This might be explained by acknowledging a synergistic effect of leucine when combined with EPA/DHA compared to being administered alone on the reduction of Ca^2+^ and tumour PTHrP. To test the potency in situations more similar to the in vivo situation, where C26 cells grow in a solid tumour in the flank of the mice, we incubated a larger number of cells with DHA or EPA, as seen in Experiment D. It should be mentioned that in vivo, the tumour consists of not only C26 cells but also stromal and inflammatory cells, which were not included in our in vitro studies. At this higher confluency, where more cell–cell contact was present, only DHA proved able to reduce PTHrP production. Previous research shows that ω3 PUFAs may exert anti-cancer activities on several different cancer types [32]. Moreover, ω3 PUFAs have been found to be pro-apoptotic and anti-proliferative in human colorectal cancer cell lines [33]. However, in our study, the effects on PTHrP production were independent of cytotoxicity or viability. One could also argue that it is unlikely that the proliferation of the cells changed, because XTT remained the same. PTHrP production in C26 cells has previously been related to COX-2 activity [8], and DHA is known to be able to inhibit COX-2 activity [12]. Therefore, our next step was to determine possible mediators of PTHrP by testing involvement of COX-2, as seen in Experiment E. To this end, we incubated a large number of cells with a specific COX-2 inhibitor CXB, and measured the effect of DHA on PGE-2 production and the effect of CXB on PTHrP and PGE-2 production. Surprisingly, DHA was not able to reduce PGE-2 production, whereas CXB was. Moreover, PTHrP production did not change upon supplementation with CXB. This was an unexpected result, since a different COX-2 inhibitor (NS-398) was able to reduce PTHrP production in C26 cells [8]. A possible explanation for the difference between our finding and the literature is that in the NS-398 experiments, PTHrP production was only present in a spheroid culture and not in monolayer cultures; in our experiments, PTHrP production was already visible in a monolayer culture. From our in vitro experiments, we can conclude that DHA is possibly the most potent component in reducing the PTHrP levels. The reduction in PTHrP production was most likely not mediated by COX-2, leaving the mechanism of action of DHA still an open question.

Bone health and calcium homeostasis are important factors affected by malignancy. In this study, we showed that tumour-induced hypercalcemia is highly correlated with several cachexia related outcomes. We also showed that a nutritional intervention high in leucine and fish oil can reduce the detrimental effects of the tumour on calcium homeostasis in a tumour-bearing C26 mouse model. This is clinically relevant, since calcium homeostasis can have a large impact on the daily life of patients. In a study of 686 multiple myeloma patients, serum calcium levels were associated with quality of life (QOL) scores, appetite loss, nausea/vomiting, physical functioning (*p* < 0.001), cognitive functioning (*p* = 0.001), and the scores for fatigue and pain (*p* < 0.001) [5]. Moreover, we elucidated possible mechanisms behind the beneficial effects of fish oil supplementation on hypercalcemia. The ω3 PUFA DHA, one of the potent components found in vivo, reduced PTHrP production of C26 cells in vitro. A reduction in PTHrP is important, independent of its effects on calcium homeostasis and bone health, since PTHrP is also related to an increase in adipose tissue browning and muscle wasting [9,34]. Further research in the mechanism involved in the beneficial effects of ω3 PUFA is needed to further optimize nutritional support for cancer patients.

## Figures and Tables

**Table 1:** Correlation of plasma calcium levels and organ weights. * and ** represent significant Pearson correlation coefficients of Experiment A (combination vs. separate compounds and controls) and B (total product vs. controls) (respectively, *p* < 0.05 and *p* < 0.01).

Pearson Correlation Coefficients with Plasma Ca^2+^ Levels	Experiment A	Experiment B
M. tibialis anterior	−0.640 **	−0.705 **
M. extensor digitorum longus	−0.498 **	−0.628 **
M. soleus	−0.579 **	−0.603 **
M. gastrocnemius	−0.623 **	−0.772 **
Epididymal fat pad	−0.659 **	−0.783 **
Spleen	0.552 **	0.695 **
Kidney	−0.409 **	−0.635 **
Liver	−0.480 **	−0.527 **
Intestine	0.243 *	0.141
Thymus	−0.701 **	−0.561 **
Heart	−0.338 **	−0.743 **
Lung	0.355 **	0.098

**Table 2:** Correlation of plasma calcium levels and muscle function of Experiment B (total product vs. controls). *, ** and # represent significant Pearson correlation coefficient intervals of parameters of EDL muscle function at the frequencies 83 till 167 Hz (* = *p* < 0.05 and ** = *p* < 0.01 for all frequencies measured; and # = *p* < 0.01 for the frequencies ≥100 Hz).

Pearson Correlation Coefficients with Plasma Ca^2+^ Levels	R Interval
Maximal force	−0.81 to −0.83 **
Maximum contraction velocity	−0.74 to −0.82 **
Maximum relaxation velocity	−0.78 to −0.85 **
Maximal force corrected for muscle mass	−0.47 to −0.53 *
Maximal contraction velocity corrected for muscle mass	−0.42 to −0.66 *
Maximal relaxation velocity corrected for muscle mass	−0.38 to −0.60 ^#^

## Supplementary Materials

The following are available online at https://www.mdpi.com/1422-0067/20/20/4978/s1.

## Abbreviations

COX-2	cyclooxygenase-2
CXB	Celecoxib
DHA	docosahexaenoic acid
EPA	eicosapentaenoic acid
IL-6	interleukin 6
PGE-2	prostaglandin E2
PTHrP	parathyroid hormone related protein
SNC	specific nutritional combination
