# Supplementary material for: A Diet Rich in Fish Oil and Leucine Ameliorates Hypercalcemia in Tumour-Induced Cachectic Mice
Source: Int J Mol Sci. 2019 Oct 9;20(20):4978. doi: 10.3390/ijms20204978 (PMC6829241; doi:10.3390/ijms20204978)
Supplement: Supplementary file 1 [file ijms-20-04978-s001.pdf]

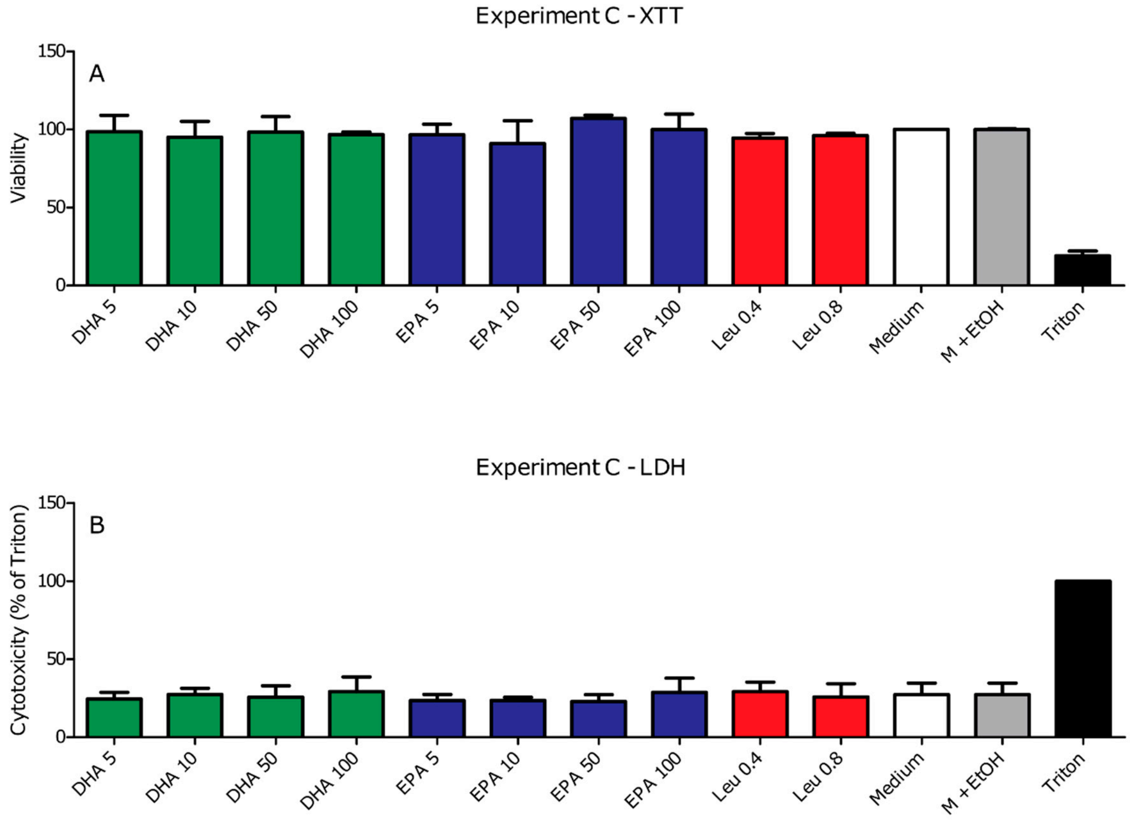

Supplemental figure 1. Measurement of viability in experiments C using XTT [A] shows that none of the experimental conditions affected viability. Measurement of cytotoxicity in experiments C using LDH [B] indicated that none of the experimental conditions was toxic to the cells.

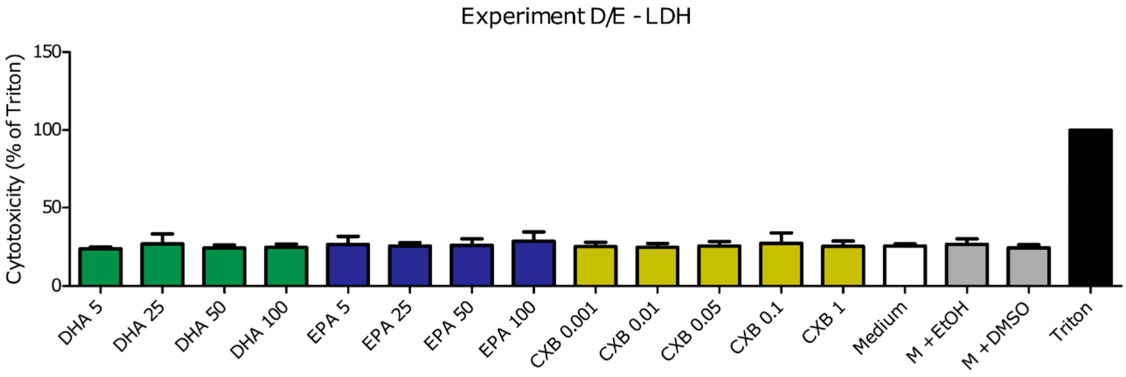

Supplemental figure 2. Measurement of cytotoxicity in experiments D and E using LDH indicated. that none of the experimental conditions was toxic to the cells.
